# Communication and role clarity inform TeleICU use: a qualitative analysis of opportunities and barriers in an established program using AACN framework

**DOI:** 10.1186/s12913-021-06287-6

**Published:** 2021-03-25

**Authors:** Anna Krupp, Michael Di Martino, Wesley Chung, Krisda Chaiyachati, Anish K. Agarwal, Ann Marie Huffenberger, Krzysztof Laudanski

**Affiliations:** 1 College of Nursing, University of Iowa, Iowa City, IA 52242 USA; 2 College of Arts and Sciences, University of Pennsylvania, 249 South 36th St, Philadelphia, PA 19104 USA; 3 Department of Chemistry, College of Arts and Sciences, Drexel University, 3141 Chestnut St., Philadelphia, PA 19104 USA; 4 The Department of Medicine, The University of Pennsylvania Perelman School of Medicine, The Leonard Davis Institute for Health Economics at the University of Pennsylvania, Penn Medicine Center for Connected Care, Philadelphia, PA 19104 USA; 5 The Department of Emergency Medicine, University of Pennsylvania Perelman School of Medicine, The Leonard Davis Institute for Health Economics at the University of Pennsylvania, Philadelphia, PA 19104 USA; 6 Penn Medicine Center for Connected Care, The Clinical Practices of the University of Pennsylvania Health System, Philadelphia, PA 19104 USA; 7 Department of Anesthesiology and Critical Care; University of Pennsylvania, JMB 127, 3620 Hamilton Walk, Philadelphia, PA 19146 USA; 8 Leonard Davis Institute for Healthcare Economics , JMB 127, 3620 Hamilton Walk, Philadelphia, PA 19146 USA

**Keywords:** TeleICU, Innovation, Workflow, Communication, Nurse, Qualitative

## Abstract

**Background:**

Understanding the use of tele-intensive care unit (ICU) services is an essential component in evaluating current practice and informing future use as the adoption and application of teleICU services expands. We sought to explore if novel ways to utilize teleICU services can emerge within an established, consulting-style teleICU model considering the program’s flexible, provider-driven operation.

**Methods:**

This was a qualitative study of one teleICU/hospital dyad using semi-structured interviews from a convenience sample of ICU (*n* = 19) and teleICU (*n* = 13) nurses. Interviews were analyzed using directed content analysis to identify themes that describe their experiences with teleICU using a deductive codebook developed from an expert consensus (American Association of Critical Care Nurses) AACN statement on teleICU nursing.

**Results:**

Three themes were identified through the qualitative content analysis: [1] nurses described *unique teleICU knowledge,* including systems thinking and technological skills, [2] the teleICU partnership supported *quality improvement* initiatives, and [3] elements of the *work environment* influenced perceptions of teleICU and its use. When elements of the work environment, such as effective communication and role clarity, were not present, teleICU use was variable.

**Conclusions:**

Flexible, provider-driven approaches for integrating teleICU services into daily practice may help define the future use of the teleICU model’s applicability. Future work should focus on the importance of effective communication and role clarity in integrating the emerging teleICU services into teleICU/ICU practice.

**Supplementary Information:**

The online version contains supplementary material available at 10.1186/s12913-021-06287-6.

## Background

Telemedicine in the intensive care unit (teleICU) is designed to support acute care for critically ill patients remotely. First developed in the 1970s as a method to achieve 24-h intensivist coverage, it is estimated that at least 15% of U.S. beds before COVID-19 had a teleICU coverage component [1, 2]. COVID-19 provided significant impetus for the acceleration and integration of these services [3, 4]. Hospitals invest in teleICU services for several reasons, mostly to provide care when resources are scarse [5, 6]. In addition to providing patient monitoring and consultation by trained ICU providers (e.g., physicians, nurses, advanced practice providers, pharmacists, and/or respiratory therapists), teleICU programs can support quality improvement programs by providing benchmarking and patient data [6–10]. Despite these applications, the financial return on investment remains unclear [5, 11]. While several single-center pre-and post-implementation analyses have shown reductions in ICU length of stay and hospital mortality, other systematic reviews and meta-analyses describe variable outcomes for hospital mortality and length of stay [12–16]. One identified challenge with evaluating teleICU benefits is that teleICU practice models vary within health care organizations and across systems. However, the process of teleICU adaptation needs to be tailored to the specific needs of the site with awareness of local culture [1, 5, 7, 12, 17, 18]. Consequently, the process of implementation may be complicated to navigate.

Establishing a robust process of matching opportunities of teleICU services to ICU demands is one of the most important goals for teleICU advancement [19–21]. Within each teleICU model, the provider roles may vary, from a supervisory role to place patient orders, a consultative role, or a co-managed partnership with ICU providers [2, 18–21]. Whereas one practice model has not been deemed as the superior approach, the method in which teleICU practice is implemented and operationalized is critical [1, 5, 7, 10, 17]. High-performing teleICU programs are successful because of leadership and organizational characteristics, not the technology’s quality [17]. These findings emphasize the importance of understanding local teleICU practice culture as both an opportunity to understand and improve the practice environment on both sides of the technology [5, 7, 11, 19–22].

Understanding the local teleICU practice environment among nurses is particularly relevant as nurses are crucial in implementing teleICU care and direct care in the ICU. Their proximity to the patient makes nurses a prime contact point for delivery and utilization of teleICU services. Beyond identifying nurses’ barriers to teleICU use, little is known about the complexity of how teleICU consultation is operationalized and how nursing workflows adapt for using teleICU services [5, 12, 21, 23]. Furthermore, guidelines for establishing and maintaining a teleICU nursing practice environment are described in clinical recommendations, but the extent to which these guidelines are present in practice is not known [21, 23, 24]. Our study could be potentially extrapolated to other fields as the AACN expert statement shares many commonalities with similar guidelines [21, 25].

In our system, the teleICU program was implemented using a flexible approach for 24/7 monitoring and consultation but with no rigidly defined roles for providers, one of the most common models [20, 21]. This arrangement may effectively foster innovation. Considering the potential high intensity for teleICU interactions, nurses can arguably be influential innovators and adaptors for effective teleICU delivery, especially when the needs are not clearly defined. Such an unconstrained ICU/teleICU model allows for addressing the care niche, which is not addressed by existing workflows. Thus, teleICU and ICU nurses’ interactions can spontaneously form the optimal healthcare delivery model organically addressing unmet needs [26]. This organic adaption of the teleICU services may answer the implementation problem and may account for the resounding success of teleICU during COVID-19 [4, 17, 18, 20–22]. We hypothesize that the teleICU program’s flexible structure will result in organic forming to perceive and integrate teleICU services, a virtually new process. A more in-depth understanding of the current implementation process may inform the future use and expansion of teleICU programs as it becomes a common way to support and provide ICU-level care, a concern frequently voiced by teleICU stakeholders [3, 8, 21].

## Methods

We conducted a descriptive, qualitative study of nursing practice regarding teleICU consultation within an established teleICU program. The University of Pennsylvania Health System instituted the PENN E-LERT® in 2004 to provide additional 24/7/365 monitoring and clinical decision support and continues to provide services to all ICU beds in the health system using physicians, advanced practice providers, and critical care nurses situated in a central teleICU unit, remote from ICU beds. This study was ruled by the Institutional Review Board at the University of Pennsylvania (#830145) as exempted from written consent since study’s purpose is quality improvement.

### Sample

Participants were registered nurses recruited from surgical ICUs within one University of Pennsylvania Health System hospital (ICU nurse) and the teleICU program (teleICU nurse). Because the teleICU provides continuous monitoring in each ICU patient room, full-time, part-time, and float staff were eligible to participate. We recruited a convenience sample of participants through public announcements, using emails and staff meetings. A total of 32 nurses participated (ICU (*n* = 19), teleICU (*n* = 13)), giving us a sufficient sample size to achieve data saturation with content analysis [27].

### Study procedure

Data were collected by interviews using a semi-structured interview guide by a trained study team member (M.D.W., W.C.) (Supplementary Material). Interviewers were not involved in the implementation or operations of the teleICU program and did not know participants. Questions of nurses focused on the following topics within teleICU/ICU nursing practice: how teleICU is currently used, acceptability, future opportunities, and perceived patient perspectives. To specifically query current practice characteristics, participants were asked to describe an example of a recent interaction with an ICU or teleICU nurse using the teleICU service. In total, we conducted 32 one-on-one interviews. All interviews were audio-recorded and transcribed verbatim using a professional service (Rev.com).

### Analysis

Data were analyzed using a directed content analysis approach to code the data [28]. An initial codebook was derived from the AACN TeleICU Nursing Practice Statement with key elements from each recommendation used to define codes [24]. The codebook was refined using an iterative process. First, each coder independently reviewed and coded the same transcript. Next, the full study team met to compare coding results, refine codebook definitions, and resolve discrepancies. For example, each code was further specified to indicate the presence or absence of any practice recommendation statement. New versions of the codebook were tested on additional transcripts until consensus was achieved on the final codebook definitions. All interviews were then coded using the final codebook.

The analysis team consisted of two trained coders involved in data collection (M.D.M, W.C), and an independent qualitative critical care nurse researcher (A.E.K.) who was not involved in data collection. Two coders independently read line-by-line sections of each transcript and assigned codes according to the predetermined themes and sub-themes in the codebook. The analysis team met weekly to discuss coding. Any discrepancies were resolved within the analysis team with input from the full study team as needed. NVivo 12© software was used for data management (QRS International, Burlington, MA) [29].

## Results

Participants’ characteristics are summarized in Table 1. We identified three main themes using directed content analysis: (1) “*continuous improvement*”; (2) “*unique teleICU knowledge*”; (3) “*collaborative work environment*” (Table 2). “*Continuous improvement*” was described as partnering to implement evidence-based practice or quality improvement initiatives and demonstrating teleICU impact. Both ICU and teleICU nurses described “*unique teleICU knowledge*” as systems thinking and technological awareness. They stressed in the same domain that teleICU enabled more proactive interventions, earlier response to urgent needs, and provided real-time decision support as compared to more conservative models. “*Collaborative work environment*” was described as tools for effective collaboration, mutual respect, and collaborative leadership. ICU and teleICU participants agreed that the teleICU provided a beneficial layer of support for patients. Despite this agreement, the proactive utilization of teleICU services was low, especially from side of the regular users. We identified several misconceptions interfering in the more widespread utilization of teleICU. The themes, sub-themes, and supportive quotes are summarized in Table 3.

**Table 1:** Self-Reported Participant Characteristics

Characteristic	ICU nurse (*n* = 19)	TeleICU nurse (*n* = 13)
Age, average	35	49
Gender, % female	57.9%	92.3%
Race, n (%)		
Caucasian	14 (73.7%)	9 (69.2%)
Asian	3 (15.8%)	2 (15.4%)
African American	2 (10.5%)	0
Other	0	2 (15.4%)
Experience, years in current position, avg. (range)	8 (2–25)	3 (< 1–12)

**Table 2:** Definitions of key themes and sub-themes derived from the AACN TeleICU Nursing Practice Recommendations^11^

AACN Recommendation and Theme or sub-theme	Definition
Collaborative work environment	Recommendation that the teleICU and health care organization establish and maintain a healthy work environment to optimize patient outcomes.
Tools for effective collaboration	Using professional communication skills, sharing goals, and having relevant patient information during teleICU and ICU interactions.
Mutual respect	Demonstrating collegial interactions, recognizing team member roles, and protecting privacy and confidentiality.
Collaborative leadership	The process of integrating teleICU and ICU practices at staff/unit and organizational levels of leadership
Unique teleICU knowledge	Recommendation that teleICU nurses establish specific knowledge and skills to contribute to nursing practice and patient outcomes.
Specialized skills	Having ICU clinical knowledge, demonstrating technological knowledge, systems thinking, and/or adaptability to adjust workflow as the environment changes.
Execution	The process of identifying proactive interventions (preventing urgent situation), identifying or responding to an urgent situation, or providing real-time clinical decision support.
Continuous improvement	Recommendation that teleICU nurses and ICUs partner in implementing best practices and measuring patient outcomes to optimize care.
Measure value	The practice of monitoring or communicating teleICU interventions or outcomes to co-workers, unit(s), and/or organizations.
Partnerships	The process of teleICU and unit collaborating to improve outcomes. i.e. collecting quality improvement data to improve patient outcomes, mentoring new clinicians to improve transition to practice.

**Table 3:** Overview of themes, subthemes, and supportive quotations

Theme or subtheme	Supportive quotations
Quality improvement	
Measure value	“We recently started a new program in a couple of the ICUs… the big question that the nurses have over there, because I round and talk to them, is what are we bringing to them? They don’t really see that there’s a benefit on their end.” TeleICU9
Partnerships	“They asked, ‘could a teleICU nurse provide a second signature?’ I said, ‘yeah, as long as we can visualize and double-check everything that you do at the bedside through the camera.’ A couple of other ideas have come from the bedside staff and we explore them.” TeleICU5 “Extubation in less than 24 h is an important metric… the [teleICU nurse] has a collaborative conversation ‘your patient is still intubated at the 18-h mark, what can be do over the next six hours to get this patient extubated?’… That’s something we’re currently utilizing [teleICU] for, which has been very helpful.” ICU1
Unique teleICU knowledge	
Specialized skills	“Having been an ICU nurse for 20+ years, your focus has to shift from being very detail-oriented for a few patients to being more generalized for a large amount of patients… I have to know a little bit about everybody and a little bit more about certain patients... you have to step back and change your approach to how you look at your patients.” TeleICU1
Execution	“We really have been able to thwart disasters at times… We visualize patients and if something is amiss, if a patient has problems with their mentation, if they’re delirious, confused, we keep an extra eye on them, and we’ll alert the staff if something is not right.” TeleICU5 “I camera’d in when the pressure was low, and I said ‘do you have access? Somebody needs to bring the cart.’ Then the doc came, and he directed the code. I was helping the nurse that was on the chest because they weren’t going fast enough. I’m like ‘you need to be 100′.” TeleICU3 “There were a few times when I wasn’t sure about something, or whether or not I should wake up the provider at night… I would call them [teleICU] and see what they thought about the situation. I maybe did that two or three times while I was working night shift.” ICU16
Work environment promotes effective collaboration	
Tools for effective collaboration	“We have to be careful on our end when we’re talking to the staff, how we modulate our voice and what words we choose to not sound condescending or fault finding. It’s an art and some people are much better at it than others.” TeleICU2 “I’ll see the camera flipped around and they’re looking at stuff, but they’re never telling me what they’re looking at or why they’re in my room. I want to know why you’re looking at my patient because if I missed something I want to know.” ICU13 “I don’t know if they don’t document it, so I’ve run into that where I’ve called and said, ‘did you do this?’ and they’re like, ‘yeah, but it’s not documented.’ So it’s frustrating on our end, but it’s frustrating on their end also.” TeleICU11
Mutual respect	“As a new nurse, it felt strange, because it felt like you were always being watched. If you change your mindset to think that it’s for helpful reasons, then it just is what it is.” ICU12 “When we have providers [in teleICU] that we know, we’re more likely to use it. It could be a big thing to know who is working over there. There are some of our former nurses who went there, so sometimes they’ll pop in and just say ‘hello’, which is nice.” ICU16
Collaborative leadership	“I feel like our [teleICU] services aren’t always advertised much. I think we need to be present on the unit more, we actually need to go to the units and do things with the nurses… it’s just that they have a different perception of us.” TeleICU3 “We monitor so many different ICUs. Each ICU has their own culture. It’s different. [Unit] was one of the first to come on board. Their physicians really started this program. They were much more receptive. Having that relationship helped a lot, where other places are not very receptive.” TeleICU7

### Theme #1 – “continuous improvement”

“*Continuous improvement*” referred to the recommendation of teleICU nurses and their bedside partners in implementing best practices while providing measures for care optimization. Implementing best practices included unit-initiated quality improvement programs with the help of teleICU. For example, to improve post-operative ventilator length of stay in a surgical ICU, teleICU nurses communicated with the ICU nurses when intubation times exceeded a specified value. TeleICU nurses also routinely described their role in supporting new-to-practice ICU nurses in implementing best practices or policies.

“*New [ICU] nurses might get something that they are not familiar with; we can help with that. I have a cardiac ICU background. A patient on a general ICU had a Swan Ganz catheter, which is something that they generally don’t get. She was unfamiliar that the catheter had floated into the right ventricle. It’s considered per policy that that’s a medical emergency and should be taken out. I talked to her about trying to prevent any issues happening until the team was able to remove it*.” TICU7.

Regardless of the improvement intervention, ICU and teleICU nurses specified a need to know the benefit of working with the teleICU for patient and program outcomes. The particular barrier emerged when several ICU nurses had the problem of describing the benefit of teleICU engagement. Conversely, the invisibility of teleICU-driven outcomes to ICU nurses was frequently reported by teleICU participants.

*“It is a job that goes on in the background. If you do not catch anything, then it is a good day. Everyone was safe, but at the same token, it is hard to quantify what we are doing.” TICU12.*

### Theme #2 – “unique teleICU knowledge”

Specialized teleICU skills include having expertise in ICU clinical knowledge and technology applications. Though technological awareness is becoming a necessary condition to be in healthcare, practicing in teleICU requires higher acumen of technology and workflow. TeleICU staff pointed out that systems thinking or seeing the big picture of patients’ needs across units was a skill learned as a teleICU nurse.

These unique teleICU skills were frequently mentioned by teleICU staff in the context of parallel monitoring of numerous patients by teleICU. This was accomplished by assessing and triaging patients quickly by taking full advantage of computerized acuity scores to determine which patients needed more monitoring.

*“Initially I’m running through everybody, looking if they are intubated or not and what their code status is, which allows me to go through this high volume of patients very quickly. And then I go back and read their progression, goals, and dig a little deeper. If somebody is a ‘green’, which is totally stable, I’ll not delve too deep. But if somebody is a ‘red’ I’ll delve deeper.” TICU4*

TeleICU nurses executed their specialized skill set in various ways, including identifying proactive interventions to prevent an urgent situation, responding to urgent patient needs, and real-time clinical decision support. There was a very high level of innovative flexibility and situational adaptation. For example, teleICU participants described contacting the ICU nurse after identifying a safety or assessment concern that could have serious patient consequences without intervention.

*“I found an alarm turned off and the patient’s blood pressure was very low, so I caught that alarm before the staff did.” TICU2.*

ICU nurses consistently described these teleICU interventions as a “second set of eyes”.

*“They're [teleICU nurse] going to call when something's not working well, or they have suggestion that we haven't tried before.” ICU4*.

In addition to patient-focused interventions, some ICU nurse participants described consulting with the teleICU nurse for a second opinion.

*“She wanted input on my end before administering an anti-hypertensive for a patient and she wasn't quite sure what was going on. I talked her through validating the arterial line, we discussed sedation, and different scenarios… she was very thankful for that.” TICU2.*

Yet, consulting the teleICU nurse was not routine practice.

*“…maybe instead of just watching the patient they [ICU nurse] could call and say, 'what do you think?', but like I said, they don't really utilize us here.” TICU3.*

### Theme #3 – “collaborative work environment”

The AACN Tele-ICU practice recommendations state that teleICU and health care organizations establish and maintain a healthy work environment to optimize patient outcomes. This is described as having tools for effective collaboration, mutual respect, and collaborative leadership. TeleICU nurses acknowledged challenges to collaboration with working remotely amid existing clinical workflows. Not being physically present in the ICU was a perceived challenge for the ability of teleICU nurses to collaborate with ICU nurses.

*"We cannot physically fix things. I think overall, it's hard even if we just got along. You know, to have someone coming in and saying, 'hey, you might want to try and do this.' Constantly when you're already busy." TICU8.*

“Fixing things” was often described as a successful intervention driven by excellent communication. Communication awareness was exhibited by teleICU during collaboration with an ICU nurse by being aware of the tone of their voice and choice of words.

*"A lot of times people snap at you when you're talking to them, but with the camera it helps if you go in there, show your face, smile, ask how they're doing. I think that can smooth over a lot of things." TICU10.*

Aside from verbal communication, teleICU nurse participants described the need for shared understanding to promote collaboration. While the electronic health record provided all clinicians the ability to view patient information, teleICU nurses described gaps in documentation that required phone calls to clarify. Several participants suggested including the teleICU nurses in rounds to promote information sharing and collaboration.

Mutual respect is achieved by recognizing team member roles and demonstrating collegial interactions. Many ICU nurse participants did not understand the teleICU nurse role and routine practice. This often was the root cause of antagonistic interactions, especially if the teleICU nurse or bedside nurse had prior conflicts.

*"I don't know the frequency of when somebody is being checked on, what they're looking for… I think people don't know that and that you can use them for anything other than just an emergency, that would be helpful." ICU7*

*"It's one of the worst parts of the job really, because it's like, 'do I call? Don't I call?' You end up feeling badly when [ICU nurses] are not receptive." TICU7.*

Sub-optimal interactions occurred because of mismatches between the perceived goals for teleICU services between ICU and teleICU nurses. The former one believes that teleICU is a backup, while teleICU nurses perceived their role to be much more.

*"Maybe it's just the unfamiliarity with it... you just kind of forget that it's there because it's kind of in the background, so I don't know. Maybe just more education about it, I think is always a good thing." ICU6*

Despite several barriers to a collaborative work environment, some teleICU and ICU nurses identified strategies for fostering a more collaborative culture. ICU participants sensed that units were more receptive to collaborating with the teleICU when there were existing relationships between the teleICU and ICU clinicians (see Table 3). Additional suggestions to facilitate integration included rounding in ICUs and sharing more data with unit and hospital leadership.

## Discussion

In our qualitative analysis of teleICU use within one teleICU/hospital dyad, we found that staff readily identified that teleICU enabled more proactive interventions, earlier response to urgent needs, and provided real-time decision support compared to more conservative models. The study demonstrated a belief that a specialized skills set is needed for teleICU practice. This observation has increasing appreciation [3, 5, 17, 21]. There was a clear need for an environment that fosters effective and clear communication and collaboration, especially when trying to implement possible teleICU implementations. Also, ICU and teleICU staff frequently expressed a desire for patient-specific outcomes that the teleICU program could impact.

Prior research has used qualitative and/or ethnographic methods to understand teleICU utilization without reference to existing patterns [17, 30]. Here, using qualitative methods to analyze data accounts for drivers of local practice factors while recognizing national practice standards by the AACN TeleICU Practice Statement, which provide criteria and common elements for developing and sustaining teleICU programs [24, 25]. The recommendations offer a standard with which to evaluate teleICU practice. Our study contributes to the understanding of factors that influence nurse teleICU use by using expert recommendations to describe practices that are present or absent and begin to understand why gaps between recommendations and practice may exist [21]. Explaining real-world experiences through the lens of national practice standards helps identify local structures and processes that might influence teleICU use but is an underutilized technique. This study evaluated whether analyzing local teleICU structures and processes inherently operationalized nursing teleICU guidelines.

Although ICU and teleICU nurses described teleICU services as necessary for improving patient outcomes and patient safety, ICU nurses’ self-reported utilization was low. ICU nurses understanding of the teleICU program was limited, which created a mismatch between ICU nurses’ needs and teleICU nurses’ expectations, a finding that is expected in a flexible program. The same mismatch likely prevents the organic growth of innovation [26]. Similar to our findings, previous qualitative and/or ethnographic studies have described how perceptions influence the use of teleICU services [22]. In a multi-site ethnographic evaluation of teleICU programs, Kahn et al. describe how perceived value is a feedback loop that can be increased through positive interactions [17]. When ICU nurse participants were able to spontaneously identify a defined role for teleICU (e.g., reducing intubation times), participants described the interaction as adding value, which is novel findings. However, this suggestion has to be translated into action. Furthermore, when bedside nurse participants described vague interactions (e.g., “a second set of eyes”), participants described negative perceptions of being watched or “hovered over.” In both cases clear communication about roles and expectations for both ICU and teleICU clinicians supports routine teleICU practice and may foster new partnerships. Clear and demonstratable initiatives provide one way to demonstrate value and improve role clarity for both new and existing teleICU programs [22]. Our study adds to the research of how perception fosters use of teleICU services. It points to potential barriers and failures for teleICU advancement and adds practical suggestions for the types of information that provide meaningful information to ICU clinicians about the value of consulting with the teleICU. It is also important to stress that communication clarity supports the two other themes – unique ICU knowledge and continuous improvement.

The absence of effective communication and/or mutual respect were frequently identified as barriers to use by both clinical roles. When there is a lack of collegial interaction, ICU and teleICU nurses described a hesitancy to communicate with each other. This finding supports previous research which found that open communication was associated with bedside and teleICU trust and satisfaction [1, 10, 12, 18]. These were suggested determinants of the ICU/teleICU program’s successful development. Interventions to improve teleICU communication have been described at the individual teleICU nurse level, such as facilitating proactive communication with the bedside nurse [17, 22]. At the organizational level, the nurse work environment is linked to patient outcomes of all kinds included mortality [31]. Future work is needed to identify and test organizational work environment interventions which facilitate and foster communication and, therefore, respect between teleICU and bedside clinicians. Differences in teleICU models and program implementation are commonly identified as potential causes for variation in teleICU outcomes studies [3, 13, 21, 24]. As our findings show, without the right work environment, the ability to establish, innovate, and grow teleICU programs will always be hampered. This finding indicates that the culture of communication is the bedrock of any potential outcome for teleICU programs.

This study has several limitations. Only nurses in surgical ICUs at one academic health system participated. Other settings, such as medical or community-based ICUs may have different results because of differences in patient needs, organizational resources, or organizational culture [10, 17]. However, our sampling approach allowed for a more in-depth understanding of nurse relationships within one tele ICU’s sustained practice model. It is also unclear if other providers like physicians would encounter similar barriers as nurses in the teleICU environment. TeleICU use may also vary depending on culture and amount of experience with teleICU [5, 11, 18, 20]. We recruited a convenience sample of nurses and captured a range of professional experience, including ICU nurses who worked prior to teleICU implementation, and ICU nurses who began practice after teleICU were implemented. The sample was also limited to the nursing staff, reducing data variance and preventing insight from other groups such as physicians, nurse practitioners, and administrators. Lastly, we recognized that social desirability bias might be present, as some study team members were teleICU clinicians. Therefore, we used trained interviewers not affiliated with the teleICU or hospital and maintained participants’ anonymity to reduce the social desirability bias.

## Conclusions

We demonstrated that communication is integral to the success of a teleICU program. Ongoing efforts to maintain communication ensures that teleICU services remain nimble in their use and application within clinical environments. Lack of understanding of each other’s roles was the most likely root cause of the communication problem and suggested avenues for potential interventions to improve communication. This feature is critical in joint ICU/teleICU environments and facilitates the use of the unique skills and continuous improvement support that teleICU programs can provide. As teleICU programs expand and grow nationally, lessons from this study will help inform the future design and implementation of teleICU programs, so patients and providers benefit.

## Supplementary Information

**Additional file 1.** “Tele-medicine – BMC Semi-structured Interview” – Provider and Patient Perspective on the Value of Direct-to-consumer Telehealth for Urgent Care: Telemedicine Provider Semi-structured Interview Guide – V4.

## Data Availability

The datasets generated and/or analysed during the current study are not publicly available due the IRB policy but are available from the corresponding author on reasonable request.

## Abbreviations

ICU: Intensive care unit
COVID: 19 – coronavirus 19
AACN: American Association of Critical Care Nurses
