# Supplementary material for: Communication and role clarity inform TeleICU use: a qualitative analysis of opportunities and barriers in an established program using AACN framework
Source: BMC Health Serv Res. 2021 Mar 25;21:277. doi: 10.1186/s12913-021-06287-6 (PMC7992609; doi:10.1186/s12913-021-06287-6)
Supplement: Supplementary file 1 — Additional file 1. “Tele-medicine – BMC Semi-structured Interview” – Provider and Patient Perspective on the Value of Direct-to-consumer Telehealth for Urgent Care: Telemedicine Provider Semi-structured Interview Guide – V4. [file 12913_2021_6287_MOESM1_ESM.docx]

**Provider and Patient Perspective on the Value of Direct-to-consumer Telehealth for Urgent Care: Telemedicine Provider Semi-structured Interview Guide – V4**

1. **Introduction**
2. **Interview Description and Permission for Taping**

Thank you for agreeing to participate in this interview. We want to understand your experiences and perspectives and therefore there are no right or wrong answers to any of the questions

This interview should take about 30 minutes. With your permission, I would like to audio record the interview because I don’t want to miss any of your comments. All responses will be kept confidential, meaning your interview responses will not be associated with you, and will only be shared with the research team. We will ensure any information included in any reports or publications later on will not identify you. You may decline to answer any question or stop the interview at any time and for any reason.

Do you have any questions so far?

May I turn on the digital recorder?

1. **Interview**
2. **Understanding of Telemedicine and Experience Providing Care**

The first set of questions will ask about your general technology usage, your understanding of telemedicine as well as your experience providing patient care using telemedicine related technology.

1. **Tell me about how you use technology and its role in your daily life.**

*Probes: Do you have a cellphone? Is it a smart phone? Do you have a personal computer? How do you use the internet? How much time do you typically spend using technology?*

1. **Tell me about the types of telemedicine services you know about.**

Thanks! Before we move on, I would like to define some terms. Because we are exploring telemedicine in the context of intensive care, the term telemedicine will refer specifically to services provided by Penn eLert. This is in comparison to “in-person” care, which refers to standard face-to-face interaction you might have during routine care for your patients.

1. **In general, what types of care do you think could be provided using tele-ICU?**
2. **Describe your experience of providing care using tele-ICU (only for tele-ICU) providers.**

*Probe: What do you expect when you have a clinical encounter using telemedicine?*

*Probe: What do you think about using telemedicine for providing urgent care*

1. **What did you like about using tele-ICU to provide care to patients?**
2. **What do you think are the limitations of providing care via tele-ICU?**
3. **If anything, what do you think patients gain, or lose, from using tele-ICU medicine?**
4. **Shaping tele-ICU**

I’d now like to ask you some general questions on telemedicine systems.

1. **What would make providers use teleICU more?**
2. **What are your thoughts on the security of patients’ information during a telemedicine visit?**

*Probe: Do you have any privacy or security concerns? Why?*

1. **What role do you think tele-ICU will play as part of your future practice?**
2. **Do you have any other thoughts on tele-ICU? Are there questions I should have asked?**
3. **Demographics**

I am now going to ask you some questions designed to help us understand who participated in the study and gather information about your background. Please feel free to answer or decline to answer as you see fit.

**What is your gender?**

**What is your age?**

**What would you describe is your current occupation/position?**

**How long have you been in that position?**

**How do you self identify in terms of your ethnicity?**

**How do you self identify in terms of your race?**

1. **Reiterate confidentiality and express thanks.**

Thank you again for taking the time to speak with me and share your experiences. Your thoughts and perspectives are helpful. I’d also like to reiterate that all your comments will be kept confidential and will not be associated with you personally in any way. Thank you for your time and thoughts.
